# The Week's Work

**Published:** 1910-07-09

**Authors:** 


### The Week's Work.

### ST. MARY'S HOSPITAL, MANCHESTER.

The new hospital for women and children recently built at Manchester cannot, although completed, be opened on account of lack of funds. It is a curious coincidence that such a deadlock has arisen once more in a hospital bearing the name of St. Mary, and it might have been thought that the example of the well-known institution at Paddington would have been sufficient warning to the managers of its Lancashire namesake to avoid at all hazards a repetition of the unfortunate Clarence wing fiasco. The new buildings have cost £50,000, but it appears that at least £5,000 guaranteed income per year must be forthcoming before they can be opened. Moreover, the expended £50,000 does not include equipment and furnishing, all of which would have to be provided before the reception of patients can possibly be commenced. Meanwhile the work done in the old premises is constantly growing; in 1909 some 4,500 maternity cases were attended, as compared with 3,988 for the previous year. Yet even to maintain the institution in Whitworth Street the income of the charity is inadequate, and the treasurer is already hinting at the necessity for curtailing the work. In these circumstances the problem of collecting funds sufficient to start the new hospital on a sound financial basis is a most difficult one, and the prospect at the moment is not any too encouraging. The fact has to be faced that Manchester people have already dipped fairly deeply into their pockets over medical charities; and the competition of the new Manchester Infirmary, the Salford Hospital, and the Ancoats Hospital —all of which have needed very substantial help lately—may have diverted funds which would otherwise have found their way into the coffers of St. Mary's. It is plain that a great effort is required, and we cannot believe that Manchester will fail to make it rather than allow the fine new buildings to remain untenanted for want of endowment. Whether the erection of these buildings on so ambitious a scale was or was not well advised is not the question now. What is done is done, and must be made the best of. We suggest to the people of Manchester that to allow their St. Mary's to stand empty, after the fashion of the namesake in London, is not making the best of things, and that they will be preserving the fair name of their city, as well as benefiting thousands of their own poor, by coming to the rescue as speedily and generously as possible.

### THE ERADICATION OF CONSUMPTION.

The Special Appeal Committee of the National Association for the Prevention of Consumption has issued its circular letter. The Association, it will be remembered, recently decided to inaugurate a special educational campaign against phthisis, and for that object a sum of at least £5,000 annually is wanted. The travelling tuberculosis exhibitions, which have already done such excellent service to the cause, are to be extended and improved, and vigorous propaganda work is to be carried on by means of lecturing caravans, which will peregrinate around the country and bring home to the people the importance of dealing with the matter as soon as possible. In addition, the Association has a scheme for regular public lectures and for the establishment of a central press bureau. We have already pointed out that there still exists a great deal of ignorance about, and prejudice against, the work that the consumption sanatoria are carrying on. This is only one of the facts which show how greatly the public is in want of education on the subject of consumption, its dangers, its prevention, and its cure. The Association, if it succeeds in winning the confidence of the public, is certain to do an immense amount of good, and its efforts should prove helpful to consumption sanatoria and hospitals as well as to the prophylactic enthusiasts. For that reason we commend its appeal heartily to the consideration of every hospital worker. One of the features of the work that the Association is trying to do is to get into touch with every man, woman, and child, and a system of sheets and cards for penny contributions has been devised which will make it possible for the poorest helper to become of really valuable assistance to the funds of the organisation. Everyone willing to help is requested to write to the Hon. Secretary, 20 Hanover Square, W. At the end of the year it is intended to issue a special letter-seal, to be sold for the profit of the Fund, and artistic postcards, designed by well-known artists, will be issued in packets and sold for the same object. The Fund will be applied solely to educational purposes, and, as the circular letter puts it, "the committee feel that the country can be aroused to a full comprehension of the enormous loss of life and money occasioned by consumption." The Earl of Derby is president of the committee, while the Duke of Devonshire and Mr. Waldorf Astor are the joint treasurers.

### SURELY A CALUMNY?

If every ebullition of sensationalism about hospitals by Yellow Press reporters were dealt with in this column, space for more serious topics would be somewhat scanty. Nor is it necessary or advisable to advertise such persons by taking them seriously. But a contributor to *Women Folk* has brought certain allegations against an unnamed London hospital that must and ought to be inquired into. Knowing what we do of the conduct of London hospitals, it is almost certain that the author has exaggerated, if no worse; but if it can be proved that this is not so, then indeed she has done well to expose a grave scandal. First, let it be said that the charges are not brought anonymously; Miss A. S. R. Martin, of Thornton Heath, is a nurse of twenty-one years' experience, according to her own statement, and should therefore know what she is talking about. Her letter appears to have been somewhat mutilated by the editorial pencil, or else her emotions obscure the exact current of her thought; at any rate, a distinct lack of consecutiveness is apparent in the arrangement of the indictment, the chief counts in which are as follows: A woman was kept waiting undressed from 9.30 to 4.30 in an out-patient department, though suffering from bronchitis and complications (*sic*). She was examined by about twenty students, had no food the whole time, and only a little water to drink, given to her in a soap-dish by Miss Martin. It must be assumed that this occurred in a general hospital, since nowhere else are twenty students likely to be found; but if the editor of *Women Folk* extends the hospitality of his columns to charges such as this, he ought to insist upon the name of the hospital being published. It is also alleged that a child was similarly kept undressed from 9 to 4.30, and when finally seen was found to have double pneumonia and pleurisy and a temperature of 107°; she was then sent back by tram and 'bus to West Norwood on a rainy day in March. Again we say we cannot believe it; the thing is incredible on the face of it, and only the fullest proof will satisfy us of its occurrence. The author goes on to some generalities about the medical profession which are both offensive and untrue. She says: "They [doctors] are one and all in for what they can make; they do not want to cure, but to experiment and prolong a case if there is money to be made." Such wild statements carry their own condemnation and incline us to put small faith in the author's credibility. The article concludes, *à propos* of nothing in particular, as follows: "The initial fee of 5s. is all to be had from the poor child, so out he goes bloodstained and giddy from the anæsthetic and loss of blood. *Murdered* slowly, but surely." It seems possible that Miss Martin is speaking of out-patient operations, though there is nothing in the context to suggest it; but what the "initial fee of 5s." refers to is even then mysterious. Why a patient of any kind who dies after an operation should be said to be murdered slowly, but surely, is also not obvious; if murdered at all, it is not slowly. These calumnies—for such they are—would not be worth notice were they not signed, and did they not contain allegations of fact; it remains for Miss Martin to prove the latter if she can.

### THE WIMBLEDON HOSPITALS.

The "crisis" respecting the two cottage hospitals at Wimbledon and the unsuccessful efforts for amalgamation has at last been settled by the decision of the Managing Committee of each declining (after prolonged negotiations) to fuse the hospitals into one centre and one interest, and to build two new hospitals, one at Copse Hill in the extreme west, and the other at Merton Rush in the south-west extremity. Strong feeling has been manifested on each side, and neither Committee would give way on certain points. The small hospital at Copse Hill, in the wealthy part of Wimbledon, having been in existence 40 years, the subscribers could not be got to throw over the sentiment of long association. The other temporary hospital, now in Merton Road, has been established ten years, and is described by the medical and nursing staffs as "falling to pieces." Though only £4,000 are yet in hand, the Committee of the latter has just paid for a site at The Rush, Merton, after having successfully objected to many other more desirable spots. Both institutions are a long way from the centre of the borough, but it is held that in course of time both will be needed by reason of the natural development of both ends of the borough. A proposal has been made that to save two competitive appeals, only one appeal be made to the public and the funds be divided. A strong Committee, led by the Mayor, has been formed to arrange a grand cricket match between the Surrey County Eleven and the Wimbledon Town Club in September for the building funds of the two new hospitals.

### COVENTRY AND WARWICKSHIRE HOSPITAL.

The Mayor of Coventry has called a public meeting to be held on July 26 in support of a scheme for supplying considerable additional accommodation at the Coventry and Warwickshire Hospital, which is greatly needed. It is announced that the speakers will include Sir Henry Burdett and Sir William Collins, M.P. It appears that the general opinion throughout the City of Coventry is that the hospital is totally inadequate at the present time to deal with the cases requiring medical attention. It would appear from the discussion at a meeting of the Coventry Board of Guardians that they have been in the habit of sending Poor Law cases requiring operation to the hospital. If this course has been taken because the Poor Law Infirmary has not contained an adequate surgical department and an adequate surgical staff, it is certainly time that the Guardians took steps to provide both in the best interests of the poor patients whom it is their special function to care for. A voluntary hospital is not the place for ordinary Poor Law cases to be sent to when requiring ordinary medical care. A voluntary hospital is intended for poor people who when well are able to maintain themselves, but who cannot bear the burden of such maintenance when they are overtaken by illness or accident. We draw attention to this distinction between the Poor Law medical cases and voluntary hospital cases, because it does not seem to have been grasped by the speakers at the Board of Guardians' meeting to which we refer.

### THE DRUG MARKET.

There have been several changes in the prices of drugs during the week, but the market continues quiet. In view of the near approach of the new opium crop, prices are about 1s. per lb. lower, but there is no eagerness to buy, as a further reduction is expected. Morphine is 2d. per ounce cheaper, but codeine is unchanged. Cocaine is tending dearer. Owing to increased demand, birch tar oil (*oleum betulae albae*) is much dearer; the demand is due to the fact that the lay press has recommended the oil for the prevention of insect bites. Cocoa butter is dearer. Buchu leaves have again advanced in price, but ipecacuanha is rather cheaper. Cantharides are dearer.

